# Children with autism spectrum disorders, who improved with a luteolin-containing dietary formulation, show reduced serum levels of TNF and IL-6

**DOI:** 10.1038/tp.2015.142

**Published:** 2015-09-29

**Authors:** I Tsilioni, A Taliou, K Francis, T C Theoharides

**Affiliations:** 1 Laboratory of Molecular Immunopharmacology and Drug Discovery, Department of Integrative Physiology and Pathobiology, Tufts University School of Medicine, Boston, MA, USA; 2 Department of Pediatrics, Athens University School of Medicine, Athens, Greece; 3 Second Department of Psychiatry, Attikon General Hospital, Athens University School of Medicine, Athens, Greece; 4 Department of Internal Medicine, Tufts University School of Medicine and Tufts Medical Center, Boston, MA, USA; 5 Department of Psychiatry, Tufts University School of Medicine and Tufts Medical Center, Boston, MA, USA; 6 Department of Integrative Physiology and Pathobiology, Sackler School of Graduate Biomedical Sciences, Tufts University School of Medicine, Boston, MA, USA

## Abstract

Autism spectrum disorders (ASDs) have been associated with brain inflammation as indicated by microglia activation, as well as brain expression and increased plasma levels of interleukin-6 (IL-6) and tumor necrosis factor (TNF). Here we report that serum levels of IL-6 and TNF were elevated (61.95±94.76 pg ml^−1^ and 313.8±444.3 pg ml^−1^, respectively) in the same cohort of patients with elevated serum levels of corticotropin-releasing hormone (CRH) and neurotensin (NT), while IL-9, IL-31 and IL-33 were not different from controls. The elevated CRH and NT levels did not change after treatment with a luteolin-containing dietary formulation. However, the mean serum IL-6 and TNF levels decreased significantly (*P*=0.036 and *P*=0.015, respectively) at the end of the treatment period (26 weeks) as compared with levels at the beginning; these decreases were strongly associated with children whose behavior improved the most after luteolin formulation treatment. Our results indicate that there are distinct subgroups of children within the ASDs that may be identifiable through serum levels of IL-6 and TNF and that these cytokines may constitute distinct prognostic markers for at least the beneficial effect of luteolin formulation.

## Introduction

Autism spectrum disorders (ASDs) are neurodevelopmental disorders characterized by impaired social interactions and communication, as well as stereotypic behaviors.^1, 2, 3, 4^ The prevalence of ASDs is estimated to be 1 in 68 children.^5^ As many as 50% of children with ASDs regress at 2–3 years old implying the involvement of some epigenetic triggers, such as high fever, infection,^6, 7^ trauma,^8^ environmental toxins^9, 10, 11^ or stress.^12^ In spite of the identification of a number of mutations in children with ASDs,^13^ its pathogenesis remains unknown. Moreover there are no objective biomarkers for either diagnosis or prognosis making effective drug development difficult.

There appear to be distinct subgroups within the ASDs, including gastrointestinal problems,^14^ mitochondrial dysfunction^15^ and ‘allergic’ symptoms,^16^ especially food intolerance and eczema.^17^ However, no group has been identified by objective biomarkers. Increasing evidence indicates that brain inflammation is important in the pathogenesis of neuropsychiatric disorders^13, 18, 19^ including ASDs. A recent paper reported microglia activation as a common finding in the brain of patients with ASDs.^20^ Microglia can be activated by mast cells (MC),^21^ which have been implicated in ASDs.^22^ In fact, the risk of ASDs appears to be 10 times higher in children with mastocytosis,^23^ a condition characterized by an increased number of activated MCs.^24^

We reported increased serum levels of the peptide neurotensin (NT) in children with ASDs.^25^ NT is a vasoactive peptide isolated from the brain^26^ and is implicated in immunity.^27^ We recently reported that serum levels of corticotropin-releasing hormone (CRH), secreted under stress, were also elevated together with NT in children with ASDs.^28^ CRH increased vascular permeability^22^ through a synergistic action with NT.^29^ Interactions among CRH, NT, microglia and MCs could contribute to brain inflammation.^30, 31^

Many children with ASDs have been reported to have ‘allergic-like’ symptoms^32^ implicating MC activation.^33^ Natural flavonoids, like luteolin and quercetin, exhibit potent anti-oxidant and anti-inflammatory activities^34^ and inhibit the release of inflammatory mediators from human MCs.^35^ Luteolin and its structurally related quercetin inhibit the release of histamine, leukotrienes, interleukin-8 (IL-8), IL-6 and tumor necrosis factor-alpha (TNF-α) from human cultured MCs^36, 37, 38^ and allergic inflammation.^39^ Moreover, luteolin inhibited IL-6 release from activated microglia^40^ and reduced maternal IL-6-induced autism-like behavioral deficits related to social interactions in mice.^41^ Luteolin also inhibits MC-dependent stimulation of activated T cells,^42^ and is neuroprotective.^43^ It also inhibits stimulation of astrocytes,^44^ as well as microglial activation and proliferation,^45, 46, 47^ protects against thimerosal-induced inflammatory mediator release from MCs ^48^ and methylmercury-induced mouse brain mitochondrial damage.^49^ One open-label clinical study showed that a luteolin-containing dietary formulation significantly improved sociability in children with ASDs.^50^

Here we report that serum IL-6 and TNF levels that were elevated in the children with ASDs in that study before treatment were significantly reduced at the end of the treatment period; moreover, this reduction strongly correlated with those children who improved by this luteolin dietary supplement.

## Materials and Methods

Fasting blood was obtained from Caucasian children (34 male and 6 female, 4–10 years of age) on the entire ASDs who participated in an open-label clinical trial conducted at the Attikon General Hospital, Athens Medical School, Athens, Greece (registered at ClinicalTrials.gov, NCT 01847521).^50^ Children were diagnosed with ASDs based on clinical assessment and corroborated by meeting the cutoff scores on both the DSM-IV-TR symptom list and the autism diagnostic observation schedule (ADOS) algorithm. They were medication free prior to blood draw for at least 2 weeks for all psychotropic medications and 4 weeks for fluoxetine or depot neuroleptics. The exclusion criteria were: (1) Any genetic condition linked to ASDs (for example, Rett syndrome, Fragile X syndrome, tuberous sclerosis or focal epilepsy); (2) Any genetic syndrome involving the central nervous system, even if the link with ASDs was uncertain; (3) Any neurologic disorder involving pathology above the brain stem, other than uncomplicated non-focal epilepsy; (4) Contemporaneous evidence, or unequivocal retrospective evidence, of probable neonatal brain damage; (5) Clinically significant visual or auditory impairment, even after correction; (6) Any severe nutritional or psychological deprivation; (7) Systemic or mastocytosis (including urticaria pigmentosa); (8) History of upper airway diseases; (9) History of inflammatory diseases (for example, juvenile rheumatoid arthritis, inflammatory bowel disease); (10) History of allergies. Informed consent was obtained from all subjects. This protocol was approved by the Ethics Committee of Attikon General Hospital, Athens Medical School, Athens, Greece.

Children were administered the dietary formulation (NeuroProtek, GMPCertified, Tishcon, Salisbury, MD, USA) containing the liposomal flavonoids (mg per capsule): luteolin (100), quercetin (70) and the quercetin glucoside rutin (30) in olive fruit extract formulated by a good manufacturing practices–certified facility (Tishcon Laboratories, Westbury, NY, USA) under contract from Algonot (Sarasota, FL, USA; www.algonot.com). Quercetin and rutin were added to the formulation as ‘decoys’ to keep the intestinal and liver enzymes occupied to allow luteolin to escape metabolism and reach the brain.

Serum was also collected from normally developing, healthy children, unrelated to the ASDs subjects, who were seen for routine health visits at the Pediatric Department of the Social Security Administration (IKA) polyclinic. Serum samples were labeled only with a code number, the age and sex of the subjects. All ASDs and control blood samples were prepared immediately and serum was stored in −80 °C. Samples were then transported on dry ice to Boston for analysis.

### Assessment of serum cytokine levels

IL-6, IL-9, IL-31, IL-33 and TNF levels were determined with commercially available enzyme-immunosorbent assay (ELISA) kits (R&D Systems, Minneapolis, MN, USA) according to the manufacturer’s protocol.

### Statistical analysis

Prior to analysis, the data were validated and inspected for outliers. The results are presented as scattergrams with symbols representing individual data points and the horizontal lines representing the mean for each group. Normality of distribution was checked with the Shapiro–Wilk’s test. Comparison between the healthy control and the ASDs groups was performed using Mann–Whitney *U*-tests. Comparison of the ASDs group at baseline and at endpoint was performed using Wilcoxon matched pair test. The effect of Vineland Adaptive Behavior Scale (VABS) domains outcome in time was investigated using a general linear model for repeated measurements. A result was considered significant at a *P*-value <0.05. The analysis was performed by using the GraphPad Prism version 5.0 software (GraphPad Software, San Diego, CA, USA).

## Results

There was no statistical difference in serum levels of IL-9, IL-31 and IL-33 between ASDs and normotypic controls (results not shown).

**Figure 1:**
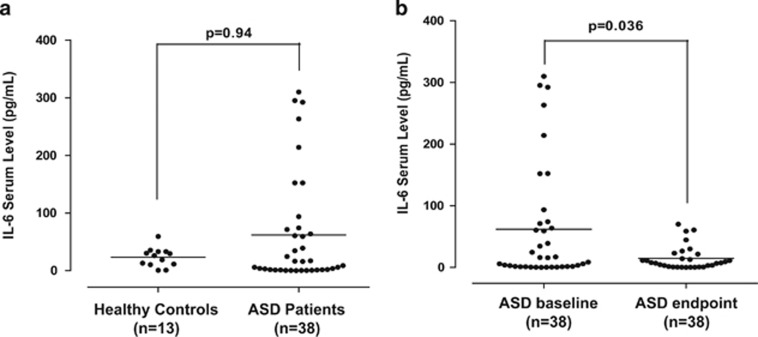
(**a**) Comparison of serum IL-6 levels in normal and ASDs children. (**b**) Serum IL-6 levels in children with ASDs before and after treatment with a luteolin-containing dietary formulation. Symbols represent individual data points, and the horizontal line represents the mean for each group. ASD, autism spectrum disorder; IL, interleukin.

Serum IL-6 levels were elevated (61.95±94.76 pg ml^−1^) in children with ASDs as compared with normotypic controls (23.20±16.31 pg ml^−1^), but this increase did not reach statistical significance (Figure 1a).

Nevertheless, serum IL-6 levels were significantly (*P*=0.036) lower (14.68±19.22 pg ml^−1^) in children with ASDs after treatment with luteolin in comparison to their levels before the beginning of treatment (61.95±94.76 pg ml^−1^) (Figure 1b).

**Figure 2:**
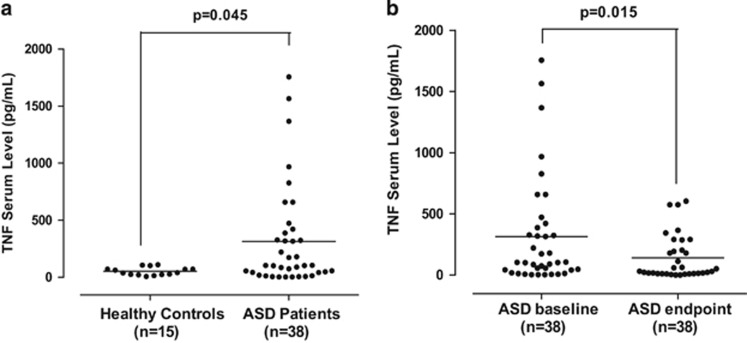
(**a**) Comparison of serum TNF levels in normal and ASDs children. (**b**) Serum TNF levels in children with ASDs before and after treatment with a luteolin-containing dietary formulation. Symbols represent individual data points, and the horizontal line represents the mean for each group. ASD, autism spectrum disorder; TNF, tumor necrosis factor.

Serum TNF levels were significantly (*P*=0.045) elevated (313.8±444.3 pg ml^−1^) in children with ASDs as compared with normotypic controls (52.78±34.62 pg ml^−1^) (Figure 2a).

These elevated serum TNF levels were significantly (*P*=0.015) lower (139.6±181.5 pg ml^−1^) in children with ASDs after treatment with luteolin in comparison to their levels before the beginning of treatment (313.8±444.3 pg ml^−1^) (Figure 2b).

There were two clusters of ASD children with low and high serum IL-6 and TNF levels indicating two subgroups. Low IL-6 and TNF levels are those below the mean, while high IL-6 and TNF levels are those above the mean. The ASDs children who had both high serum IL-6 and TNF levels were the same (*n*=10).

The improvement in the VABS age-equivalent scores for these 10 ASDs children was significant (*P*<0.05) for all domains (Table 1). The VABS composite score was also significantly higher at the end of the study. These data indicate a positive effect of the luteolin dietary supplement on the adaptive functioning of this subgroup of ASDs children. More specifically these children gained 9.73 months in the communication domain, 6.64 months in daily living skills and 8.09 months in the social domain.

## Discussion

Our study shows two clusters of ASD children with low and high serum IL-6 and TNF levels, indicating two subgroups. Moreover, the ASD children who had both high serum IL-6 and TNF levels were the same (*n*=10). We further show that the children with ASDs in which the elevated serum IL-6 and TNF levels decreased at the end of the treatment period with a luteolin formulation, were the ones whose behavior improved the most.

There is evidence indicating that certain cytokines can impair neurodevelopment and behavior^13, 51^ and that microglia activation and inflammation is involved in the pathogenesis of neuropsychiatric diseases^13, 51^ including ASDs.^52, 53^ IL-6 can directly alter neuronal activity, proliferation and survival that may impact behavior.^54^ IL-6 has also physiological and pathological effects on learning and memory.^55^ Increased gene expression of IL-6 was noted in postmortem specimens of the temporal cortex of the brain of individuals with ASDs^56^ and increased protein level of IL-6 was found in the brain and cerebrospinal fluid of individuals with ASDs.^57^ In agreement with these findings, IL-6 was significantly increased in the frontal cortex and cerebellum of ASD patients as compared with the age-matched controls.^58^ IL-6 may derive from microglia cells, which are activated in ASDs.^20^ Consistent with our results is a previously reported meta-analysis of increased IL-6 concentrations in peripheral blood in ASD participants compared with healthy controls.^59^ It is of interest that acute restraint stress of mice led to increased serum IL-6, which was entirely MC dependent.^60^ IL-1β can stimulate selective release of IL-6 from MCs.^61^

In the present study, we also found significantly increased serum TNF levels in children with ASDs in comparison to healthy controls. Another study showed increased TNF production in peripheral blood mononuclear cells of autistic subjects after stimulation with polyhydroxyalkanoates and tetanus.^62^ TNF was increased almost 50 times in the cerebrospinal fluid of ASD children.^63^ Brain MCs can secrete TNF.^64^ Both TNF and IL-6 expression has been documented in brains of children with ASDs^65^ and IL-6 has been implicated in an animal ‘model’ of autism.^7^ In this context, it is particularly important that MCs are the only cells that store preformed TNF, which they can secrete rapidly.^66^ MCs are the only cells that release IL-6 in response to stress.^60^ Preformed TNF is secreted from MCs^67^ and stimulates T-cell activation.^42, 68^ TNF has been linked with neurite growth and the regulation of homeostatic synaptic plasticity in the hippocampus.^69^

One study also showed increased serum levels of IL-17 in children with ASDs.^70^ Another study showed increased plasma IL-1β and IL-17, but only in children with ASD and regression; children with ASD and GI issues had higher plasma IL-1β and IL-6, but not TNF. TNF and IL-17 seem to act together in perpetuating the inflammatory process.^71, 72^ MC-derived IL-6 and transforming growth factor beta (TGFβ) induce the development of Th-17 cells through dendritic cell maturation;^73^ Moreover, MCs secrete IL-17, themselves.^74^

Here we report that treatment with a luteolin-containing dietary formulation normalized serum IL-6 and TNF in those children that showed the most benefit from the use of luteolin.

We recently reported that the structural analog of luteolin 3′,4′,5,7-tetramethoxy flavone was more potent than luteolin in its ability to inhibit mediator release from human MCs.^35^

Luteolin is structurally closely related to 7,8-dihydroflavone, which was shown to have brain-derived neurotrophic factor (BDNF)-like activity.^75^ In fact, absence of BDNF was associated with autistic-like-behavior in mice, while 7,8-dihydroflavone administration reduced symptoms in a mouse model of Rett syndrome,^76^ most patients with which have symptoms of ASDs.^77^

We believe this is the first time that objective biomarkers can (a) distinguish a subgroup of children with ASDs and (b) their reduced level correlate with a favorable clinical outcome, following administration of a natural anti-inflammatory compound. Flavonoids are considered generally safe^78, 79, 80^ and are being discussed as possible treatment of central nervous system disorders^81^ that may involve brain inflammation in response to environmental triggers. One obvious question is how much of the luteolin may reach the brain because flavonoids purely absorb orally and are extensively metabolized.^82, 83, 84^ Unfortunately, children with ASDs are prescribed many other supplements and psychotropic drugs that may have unwanted drug interactions.^85^ One way to deliver luteolin directly to the brain would be through intranasal administration through the cribriform plexus as shown before for another compound.^86^

## Figures and Tables

**Table 1:** Effectiveness of the study luteolin formulation in ASDs children with high serum IL-6 and TNF levels (*n*=10)

*Vineland adaptive behavior scales (VABS)*	*0 week*	*26 week*	*Change*	*Effect size^a^*	P*-value*
Communication AE	38.91 (25.47)^b^	48.64 (31.60)	9.73	0.38	0.008
Daily living skills AE	38.45 (19.11)	45.09 (21.00)	6.64	0.35	0.0003
Social AE	36.55 (18.04)	44.64 (20.93)	8.09	0.45	0.001
Composite score	37.97 (19.68)	46.12 (23.19)	8.15	0.42	0.001

Abbreviations: AE, age equivalent; ASD, autism spectrum disorder; IL, interleukin; TNF, tumor necrosis factor; VABS, Vineland Adaptive Behavior Scale.

a (26 week–0 week)/0 week s.d.

b VABS scores (s.d.).
